# Changes in Gene Expression and Metabolite Profiles in *Platanus acerifolia* Leaves in Response to Feeding Damage Caused by *Corythucha ciliata*

**DOI:** 10.3390/ijms20143465

**Published:** 2019-07-15

**Authors:** Fengqi Li, Chunyan Wu, Youssef Dewer, Du Li, Cheng Qu, Chen Luo

**Affiliations:** 1 Institute of Plant and Environment Protection, Beijing Academy of Agriculture and Forestry Sciences, Beijing 100097, China; 2 College of Life Sciences, Capital Normal University, Beijing 100037, China; 3 Bioassay Research Department, Central Agricultural Pesticide Laboratory, Sabahia Plant Protection Research Station, Agricultural Research Center, Alexandria 21616, Egypt

**Keywords:** *Platanus acerifolia*, *Corythucha ciliata*, RNA-seq, differentially expressed genes (DEGs), metabolites

## Abstract

The sycamore lace bug, *Corythucha ciliata* (Say) is a highly invasive pest insect that feeds on sycamore trees (*Platanus* spp.) worldwide. The interaction between *Platanus* species and this insect pest has not yet been studied at the molecular level. Therefore, a recent study was conducted to compare the gene expression and metabolite profiles of *Platanus acerifolia* leaves in response to *C. ciliata* feeding damage after 24 and 48 h. We employed high throughput RNA sequencing (RNA- seq) to identify a total of 2,828 significantly differentially expressed genes (DEGs) after *C. ciliata* feeding. In addition, 303 unigenes were found to be up-regulated at both time points. Moreover, Kyoto Encyclopedia of Genes and Genomes (KEGG) pathway enrichment analysis showed that monoterpenoid biosynthesis, the linoleic acid metabolism pathway, and alpha- linolenic acid metabolism were the most prominent pathways among the DEGs. Further analysis of the metabolite profiles showed that nine metabolites were significantly different before and after *C. ciliata* damage. In addition, we analyzed DEGs detected in the *P. acerifolia* and *C. ciliata* interaction using Mapman. The terpene synthase gene family was also identified. We suggest that the results obtained from DEGs and metabolite analysis can provide important information for the identification of genes involved in the *P. acerifolia*–*C. ciliata* interaction, which might be necessary for controlling *C. ciliata* efficiently.

## 1. Introduction

*Platanus* L. is an important genus of large trees commonly known as sycamore or plane trees. *Platanus* species are widely planted all over China as shade trees due to their large spreading crown, and also for their rapid growth and pruning resistance. The sycamore lace bug, *Corythucha ciliata* (Say, 1832) (Hemiptera: Tingidae), which is native to the central and eastern parts of North America, was subsequently introduced into Europe and Asia [1,2]. This pest has a strong ability to spread and is considered to be a new alien invasive insect pest in China [2]. *C. ciliata* specifically damages members of the *Platanus* genus, including *Platanus occidentalis*, *P. acerifolia*, and *P. orientalis*. The most important relationship between *P. acerifolia* and *C. ciliata* is that *P. acerifolia* is the most widely planted *Platanus* species in China [3]. *C. ciliata* was first discovered in Changshan, China in 2002. So far, *C. ciliata* has been observed to cause damage to *P. acerifolia* trees in Hunan, Hubei, Shanghai, Zhejiang, Jiangsu, Shandong, Henan, Chongqing, Guizhou, and Beijing in China [2,4], and it has been included in the list of forest pests in China. *C. ciliata* infestation can cause growth disruption, weakness, and even death in *P. acerifolia*, and it has seriously affected landscaping in many Chinese cities. Currently, the management of *C. ciliata* still mainly depends on chemical insecticides [5]. However, the *P. acerifolia* is a widely planted street tree in urban areas, so the use of pesticides does not only cause serious environmental pollution but could harm human health. Therefore, to understand the interaction mechanism between *P. acerifolia* and *C. ciliata* would provide a great help to accelerate the development of an effective environmentally friendly pest management strategy to manipulate the populations of *C. ciliata* which is also problematic for other Asian and European countries. 

Unlike animals, higher plants, which are sessile, cannot escape from the surroundings, and adapt themselves to the changing environments by a series of molecular responses aimed to cope with these challenges. The physiological basis for these molecular responses is the integration of many transduced events into a comprehensive network of signaling pathways. Consequently, plants have developed various defense strategies to respond to insects feeding on them [6,7]. These include changes in hormone levels, production of terpene volatiles that can affect insect behavior, and the production of flavonoid secondary metabolites [8,9]. In this way, plants enhance their resistance to pests. In a word, it is at the molecular level–gene expression and the control in time and space that we can bring out the mystery of living organisms and explore the nature of environmental changes.

In order to study the molecular biology of the *P. acerifolia* and *C. ciliata* interaction, *C. ciliata* were allowed to feed individually on the leaves of *P. acerifolia*, and then we combined RNA-seq and metabolite analysis to study the response of *P. acerifolia* to *C. ciliata* damage at the gene expression level. Differences in *P. acerifolia* leaf metabolite profiles were detected using metabolomic techniques. Finally, the key genes responsible for defense against this pest, the possible signaling pathways, and defensive compounds were identified. The results of our study provide basic knowledge for designing novel strategies to control *C. ciliata* in the future.

## 2. Results

### 2.1. Transcriptome Sequencing and RNA-seq Read Assembly

The newly emerged *C. ciliata* adults were allowed to feed individually on *P. acerifolia* leaves for 24 and 48 h. RNA was then extracted from the leaf tissue samples (three biological replicates at each of two different time points after infestation of *C. ciliata* to *P. acerifolia* leaves). The control group was represented by leaves not infested by insects. Also, 12 cDNA libraries were constructed for RNA sequencing. The sequencing data is stored in the NCBI Sequence Read Archive (SRA, http://www.ncbi. nlm.nih.gov/Traces/sra); under BioProject ID number PRJNA484863 and accession numbers SRR8631807 to SRR8631818. High throughput DNA sequencing generated 7.08 × 10^9^ to 8.66 × 10^9^ raw bases per sample, of which >93% had Q-scores between 20 and 30 (Table 1). The total length of the combined reads from the 12 samples was 2.02 × 10^8^ bp, and there were 199,080 unigenes with an average length of 1015.15 bp and N50 of 1579 bp (Table 2). The Mean reads mapped to the trinity reference assemble were 1442.764bp.

### 2.2. Identification of DEGs in Response to C. ciliata Feeding Damage

**Figure 1:**
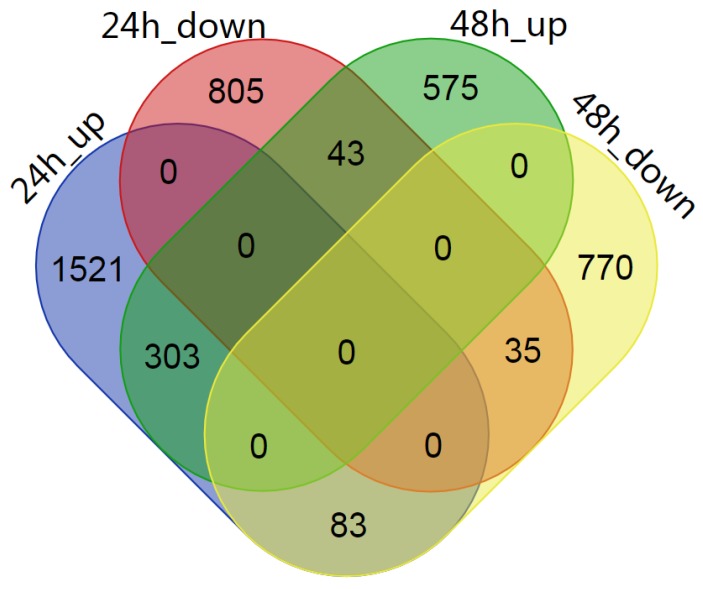
Venn diagram showing the DEGs in *P*. *acerifolia* leaves in response to *C. ciliata* damage after 24 and 48 h of feeding.

To analyze the gene expression patterns induced by *C. ciliata* feeding in the 24 and 48 h periods, we further examined the differential gene expression by comparing gene expression values (Transcripts Per Million, TPM) between the feeding treatments vs. the controls at each time point. A p-adjust value ≤0.05 and |log2 fold change |≥1 were set as the thresholds to detect significant DEGs. A total of 2790 DEGs was identified between the 24 h treatment and the control (Figure 1). Among these, 1907 unigenes were up-regulated, and 883 unigenes were down-regulated. Furthermore, 1809 unigenes (921 up-regulated and 888 down-regulated) were expressed differentially after the 48 h treatment (Figure 1). In addition, we analyzed the DEGs that overlapped between these two treatment time points. The results revealed that there were 303 and 35 up- and down-regulated DEGs that overlapped in the 24 h and 48 h treatments, respectively (Figure 1). Also, 2326 and 1345 DEGs were distinctive in the 24 h and 48 h treatments, respectively (Figure 1).

### 2.3. GO Enrichment Analysis of the DEGs

**Figure 2:**
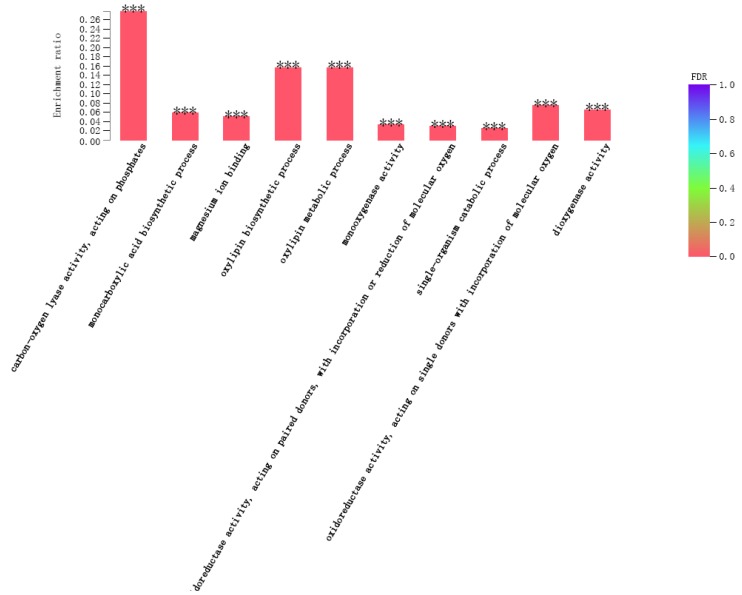
Top ten GO terms that were enriched in the 303 unigenes that were up-regulated at both time points. The x-axis represents the GO term and the y-axis represents the enrichment ratio (the number of unigenes annotated to a GO term in a gene set divided by the number of unigenes annotated to this GO term in the entire unigenes as background set.) The color indicates the significance of the enrichment. *** mean False Discovery Rate <0.001.

To investigate the functions of the DEGs detected in the *P. acerifolia* response to *C. ciliata* feeding, we analyzed the DEGs by Gene Ontology (GO) term enrichment. For the 24 h treatment, the significantly enriched GO terms in the DEGs, 159 GO terms were significantly enriched, the ten most significant terms were: oxidoreductase activity, acting on single donors with incorporation of molecular oxygen, incorporation of two atoms of oxygen (GO:0016702), dioxygenase activity (GO:0051213), monocarboxylic acid biosynthetic process (GO:0072330), lipid biosynthetic process (GO:0008610), terpene synthase activity (GO:0010333), fatty acid metabolic process (GO:0006631), organic acid biosynthetic process (GO:0016053), carboxylic acid biosynthetic process (GO:0046394), monooxygenase activity (GO:0004497), heme binding (GO:0020037) (Table S1 and Figure S1). In the 48 h treatment, 187 GO terms were significantly enriched, the 10 most significant terms were: protein-chromophore linkage (GO:0018298), cell wall (GO:0005618), external encapsulating structure (GO:0030312), glucosamine-containing compound catabolic process (GO:1901072), glucosamine- containing compound metabolic process (GO:1901071), chitin metabolic process (GO:0006030), amino sugar catabolic process (GO:0046348), chitin catabolic process (GO:0006032), aminoglycan metabolic process (GO:0006022), aminoglycan catabolic process (GO:0006026) (Table S2 and Figure S2). Also, for the 303 unigenes that were up-regulated at both time points, 86 GO terms were significantly enriched, the ten most significant terms were: carbon–oxygen lyase activity, acting on phosphates (GO:0016838), monocarboxylic acid biosynthetic process (GO:0072330), magnesium ion binding (GO:0000287), oxylipin biosynthetic process (GO:0031408), oxylipin metabolic process (GO:0031407), monooxygenase activity (GO:0004497), oxidoreductase activity, acting on paired donors, with incorporation or reduction of molecular oxygen (GO:0016705), single-organism catabolic process (GO:0044712), oxidoreductase activity, acting on single donors with incorporation of molecular oxygen (GO:0016701), dioxygenase activity (GO:0051213) (Table S3 and Figure 2).

### 2.4. KEGG Enrichment Analysis of the DEGs

We used KEGG pathway analysis to map the DEGs to the biochemical pathways involved in the plant response to *C. ciliata* damage. “Monoterpenoid biosynthesis”, “Linoleic acid metabolism” and “alpha-Linolenic acid metabolism” were prominent pathways between the treatments and controls in response to *C. ciliata* feeding damage after 24 and 48 h (Tables S4 and S5). In addition to these three pathways, “Phenylpropanoid biosynthesis”, “Phenylalanine, tyrosine and tryptophan biosynthesis”, “Flavonoid biosynthesis”, “Terpenoid backbone biosynthesis”, and “Sesquiterpenoid and triterpenoid biosynthesis” are five of 26 pathways that were enriched at 24 h (Table S4). In the 48 h treatment, 22 pathways were enriched, including “Photosynthesis-antenna proteins”, “Photosynthesis”, “MAPK signaling pathway-plant”, “Cutin, suberine and wax biosynthesis”, and “Pentose and glucuronate interconversions” (Table S5). For the 303 unigenes that were up-regulated at both timepoints, there were 17 enriched pathways. Of these 17 pathways, the first three prominent pathways of significance are “Monoterpenoid biosynthesis”, “Linoleic acid metabolism pathway” and “alpha-Linolenic acid metabolism” (Table S6). The results of the above analyses indicate that these pathways may be involved in the interaction between *P. acerifolia* and *C. ciliata*.

### 2.5. MapMan Overview of DEGs Related to Plant–Pest Interaction

**Figure 3:**
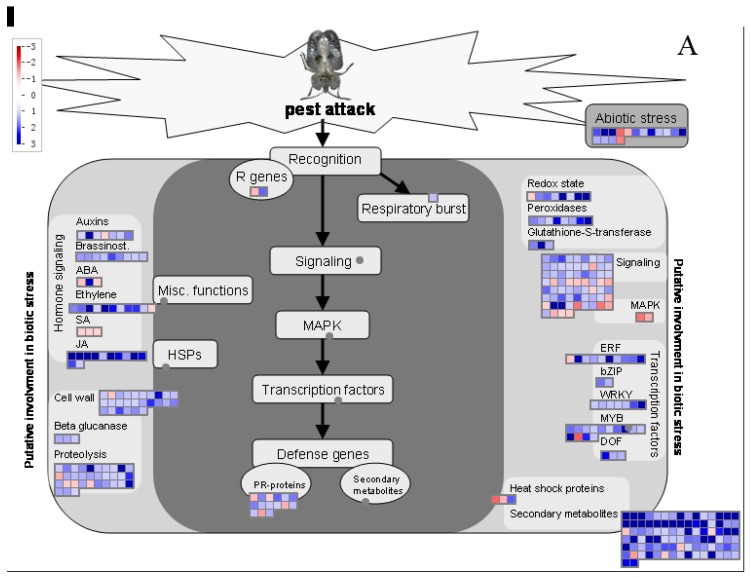

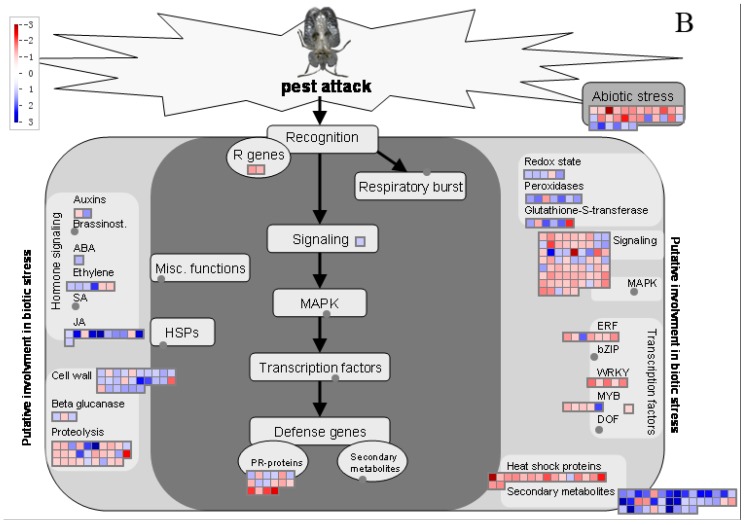
MapMan visualization of DEGs in the *P. acerifolia* response to *C. ciliata* feeding damage. The up- and down-regulated genes are indicated in red and blue colors, respectively. The log_2_FC (treatment TPM/mock TPM) are shown in the scale bars. (**A**) 24-h treatment. (**B**) 48-h treatment.

To reveal the role of DEGs in the plant response to *C. ciliata* feeding damage, we used the MapMan package to investigate the *P. acerifolia* biotic stress pathways. An overview of the role played by biotic stress in the regulation of DEGs, such as hormone signaling genes, Transcription Factors (TFs), Mitogen-Activated Protein Kinases (MAPK), Pathogenesis-Related (PRs), and PR-proteins, is shown in Figure 3. After 24 h of feeding, most of the DEGs belonging to JA pathways were up-regulated, as were most of the WRKY, myeloblastosis (MYB), and Ethylene Responsive Factor (ERF)-related DEGs, while all DEGs involved in SA signaling were down-regulated (Figure 3A). After 48 h, one ABA-related DEG and most of the DEGs belonging to redox state, peroxidase, and glutathione-S-transferase pathways were up-regulated, while one most of the DGEs belonging to known TF families such as WRKY, ERF, and DNA-binding with one finger (DOF) and two of the three heat shock proteins genes were down-regulated (Figure 3B). A detailed list of all the DEGs involved in the *P. acerifolia*–*C. ciliata* interaction and their MapMan functional categories is given in Table S7.

### 2.6. Response of Terpene Synthase Gene Expression to C. ciliata Damage

Terpene synthase genes catalyze the biosynthesis of terpenoid volatiles in *P. acerifolia* leaves. We identified 14 putative terpene synthase (TPS) genes from our RNA-seq dataset. Among them, seven showed significantly up-regulated expression after both 24 and 48 h of *C. ciliata* feeding. These up- regulated TPS genes may be involved in enhancing resistance to *C. ciliata* by producing volatiles that either repel this pest or attract its natural enemies. To examine the phylogenetic relationships among these TPS proteins and those from several other species, we constructed an unrooted neighbor-joining phylogenetic tree. The TPS genes were classified into five different families (clades) named TPS-b to TPS-g (Figure S3 and Table S8).

### 2.7. Metabolite Changes in *P. Acerifolia* Leaves in Response to *C. ciliata* Feeding Damage

**Figure 4:**
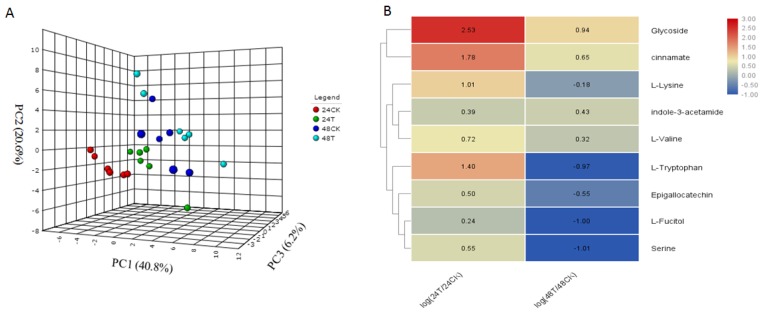
PLSDA and the changes in metabolite profiles in *P. acerifolia* leaves in response to *C. ciliata* feeding damage. (**A**) PLSDA of 50 metabolites. (**B**) Changes in nine significantly different metabolites between the 24 and 48 h treatments and controls. The numbers in the heat map represent the log values of the ratios between treatments and controls.

**Figure 5:**
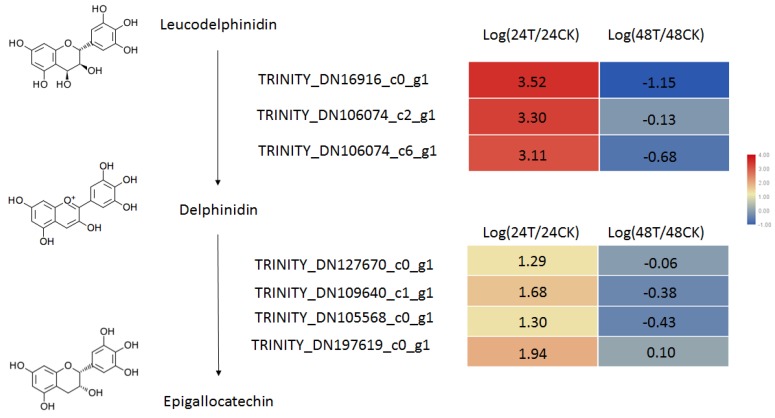
Candidate genes in the epigallocatechin biosynthesis pathway. The numbers in the heat map represent the log values of the ratios between the treatments and controls.

To analyze the changes in metabolite profiles that occur in response to *C. ciliata* feeding, we compared the metabolic compounds isolated from *P. acerifolia* leaves after *C. ciliata* adults were allowed to feed for 24 and 48 h. We detected 50 metabolites in these samples and performed Principal component analysis (PCA) (Figure S4) and Partial least squares discriminant analysis (PLSDA) (Figure 3A) analyses. As shown in Figure S4 and Figure 4A, the *C. ciliata* feeding treatments and the controls can be distinguished by their metabolite profiles. After ANOVA analysis, we found that the levels of nine metabolites were significantly different (p <0.05); these included glycoside, cinnamate, indole- 3- acetamide, L-tryptophan, epigallocatechin, L-fucitol and three amino acids (L-lysine, L- valine, and serine) (Figure 4B). Flavonoids are often closely related to insect interactions in plants. In this study, we detected one flavonol chemical, epigallocatechin, which is increased at 24 h and decreased at 48 h. Combining the RNA-seq datasets, we detected seven key genes for epigallocatechin biosynthesis, including three genes involved in the biosynthesis of delphinidin from leucodelphinidin, and four genes that catalyze the biosynthesis of epigallocatechin from delphindin (Figure 5). In addition, the relative expression values of these genes are consistent with the observed changes in epigallocatechin levels (Figure 5). These genes should be candidates for the key genes in the epigallocatechin biosynthesis pathway. 

### 2.8. Validation of RNA-seq Results by qRT-PCR

**Figure 6:**
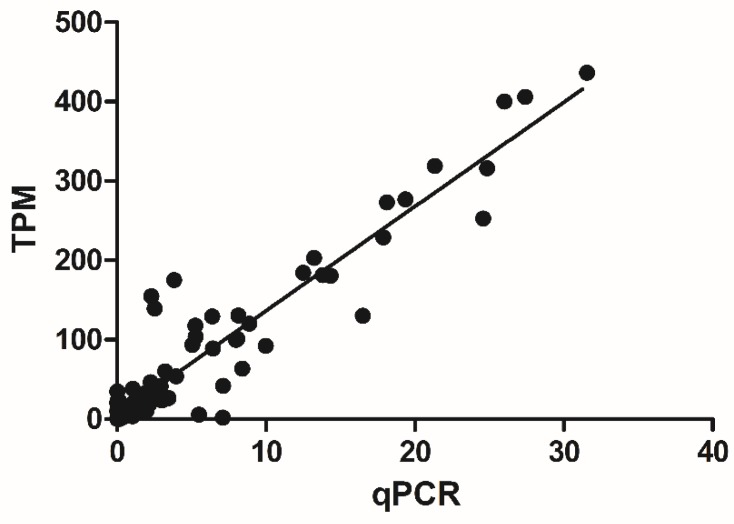
Correlation analysis between gene expression values obtained from RNA-seq and qRT-PCR. The value of qRT-PCR (x-axis) were plotted against RNA-seq values (y-axis).

To validate the unigene TPM values obtained by RNA-seq, the expression of 32 unigenes was tested using qRT-PCR assays. The primers were listed in Table S9. These unigenes included TPS genes, and genes for heat shock proteins, peroxidases, and ethylene-responsive transcription factors. We observed a strong significant correlation between the RNA-seq TPM values and the qRT-PCR expression data (Pearson’s r correlation coefficient = 0.905, p < 0.01) (Figure 6). These results shown that the gene expression profiles determined by RNA-seq in this study are reliable.

## 3. Discussion

The sycamore lace bug, *C. ciliata*, is an important insect pest that has been causing damage to *P. acerifolia* trees in recent years [2]. *C. ciliata* usually feeds on the undersides of *P. acerifolia* leaves, and the leaves develop white spots at the beginning of feeding at the upper side that can cause the leaves to appear chlorotic. The damage caused by this insect pest can reduce leaf photosynthesis, leading to a decline in some physiological and biochemical indexes such as leaf photosynthetic rate, stomatal conductance, transpiration rate, chloroplast pigments and soluble sugars. *C. ciliata* can only complete its life cycle on the sycamore plant. Based on the importance of the host plant and the limited host specificity of this pest, studying the molecular mechanisms behind the *P. acerifolia*– *C. ciliata* interaction is important for understanding the expression of resistance in *Platanus* species to this insect pest. 

By analyzing gene expression profiles at two different time points, we identified 1907 and 921 unigenes that were significantly up-regulated after 24 and 48 h of feeding, respectively. Previous studies have shown that insect stress can induce massive changes at the transcriptional level in some plants [10,11,12]. These changes can reprogram primary and secondary metabolic pathways, activating defense genes and signaling pathways to respond to insect attack. The large number of genes that showed changes in expression in our transcriptome are consistent with these findings. The results of our study establish a signature for *C. ciliata*-induced changes to the leaf transcriptome of *P. acerifolia*. 

A total of 303 genes were up-regulated in leaves after both 24 and 48 h of *C. ciliata* feeding. These 303 genes are the key core genes expressed during the interaction between *P. acerifolia* and *C. ciliata*. Combining the GO, KEGG enrichment analysis, and MapMan results, we speculated that JA-related pathways and terpenoids play an important role in this plant–insect interaction system. The plant hormone JA plays vital roles in regulating the plant response to various insect stresses [13]. In our experiments, JA-related genes were induced in response to *C. ciliata* feeding damage. This indicates that in *P. acerifolia*, JA signal transduction may act through a mechanism similar to that in other plants. At the same time, in addition to JA pathway-related genes, we also identified related functional genes, especially genes that encode peroxidases and a series of transcription factors. The identification of these genes will provide the basis for the subsequent development of insect-resistant *Platanus* trees through genetic screening and possibly through genetic transformation.

The controlled production of plant volatiles plays an important role in the indirect plant defense response against insects [13]. Following pest damage, plants will release a series of volatiles, such as terpenes, to repel the insect pests and attract natural enemies, thereby improving their insect resistance to protect themselves [7]. In our previous study, we evaluated the types and contents of volatiles in leaves of *P. acerifolia* damaged by *C. ciliata* [6]. Four chemicals, including cis-3-hexen-l-ol, 1,8-cineole, (*E*)-4,8-dimethyl-1, 3,7-nonatriene, and trans-β-caryophyllene were detected in undamaged leaves. In addition, another five chemicals, α-thujene, sabinene, myrcene, cis-3-hexenyl acetate, and γ-terpinene, were induced in leaves after feeding by *C. ciliata*. Among these volatile chemicals, cis-3-hexen-1-ol and 1,8-cineole were found to significantly attract *C. ciliata*, whereas trans- β-caryophyllene repelled this insect pest [14]. Our study further identified 14 genes involved in the biosynthesis of these compounds. Seven genes showed significant up-regulation at both 24 and 48 h after feeding. These genes are candidate synthase genes for the above terpene volatiles. However, their function needs to be confirmed by protein expression and purification, in vitro enzymatic assays using geranyl diphosphate (GPP), farnesyl diphosphate (FPP), and geranylgeranyl diphosphate (GGPP) as substrates, and GC-MS analysis.

Plant secondary metabolites also play important roles in plant-insect interactions [15]. Insects often identify these metabolites by taste to determine whether the plant can provide the corresponding nutrient resources. At the same time, plants can also negatively affect insect digestion and development by producing certain secondary metabolites, thereby achieving functional insect resistance [16,17,18]. Flavonoid secondary metabolites are particularly important in this type of interaction [17,18]. In this study, we found that the epigallocatechin content increased significantly after 24 h of feeding, although it had decreased by 48 h. Previous studies on epigallocatechin have shown that this compound is involved in plant resistance to diseases [19]. Considering that the interaction between plants and phloem-feeding insects has many similarities with the resistance of plants to pathogens [20,21], we hypothesize that epigallocatechin plays an important role in the interaction between *P. acerifolia* and *C. ciliata*. We also identified the candidate key enzymes involved in the biosynthesis of this compound by analyzing our leaf transcriptome dataset. Functional studies on the proteins encoded by these candidate genes need to be performed to determine their roles in epigallocatechin biosynthesis. A biological study should be carried out to observe the effects of epigallocatechin on feeding, development, and oviposition by *C. ciliata*.

Our study examined the intrinsic mechanisms underlying the interaction between *P. acerifolia* and *C. ciliata* using transcriptome sequencing and metabolite analysis. The high-throughput RNA sequencing technology used in this study provides a comprehensive look at the transcriptome and does not rely on the limited genomic resources presently available for *Platanus* species. It is worth noting that this study was performed on detached leaves and this could not fully represent the response of leaves on the tree under natural conditions. In the future, similar studies using leaves under natural conditions are needed to further confirm the key candidate genes and compounds found in the study. Additionally, further functional research on DGEs identified here should be carried out in order to clarify their specific roles in plant–insect interactions, thereby enabling the development of insect-resistant sycamore trees and providing a basis for the development of strategies to protect sycamore trees and to manage *C. ciliata* infestations.

## 4. Materials and Methods

### 4.1. Plant Materials and Insects

*Corythucha ciliata* nymphs were collected from *P. acerifolia* trees planted at the Beijing Forestry University in Haidian District, Beijing. The nymphs were raised indoors on fresh *P. acerifolia* leaves at room temperature (25 °C ± 2 °C), a relative humidity of 50%–70%, and a 16 h day/8 h night photoperiod. The *P. acerifolia* leaves used in the experiments were collected from the Institute of Plant Protection of the Chinese Academy of Agricultural Sciences in Haidian District, Beijing. Only healthy and untreated leaves were selected for the experiments. The leaves were removed from the tree by cutting the base of the petiole with a sterile scalpel, and they were inserted into narrow glass test tubes containing 10 mL sterile water. After the insertion of the leaf, the petioles were sealed with absorbent cotton and placed in a 2 L glass beaker. One-hundred newly-emerged *C. ciliata* adults were placed on each leaf. The experiments were carried out in artificial climate chambers (the experimental conditions were the same as the above-mentioned insect feeding conditions). The measurements in three biological replicates at each of two different time points after infestation of *C. ciliata* to *P. acerifolia* leaves (24 and 48 h) were performed. The control group was represented by leaves not infested by insects. After the feeding periods were finished, the insects on the leaves were removed with a soft brush. RNA was extracted from the leaves for RNA-sequencing and qRT-PCR assays.

### 4.2. RNA-Sequencing and qRT-PCR

Total RNA was isolated from plant tissues (leaves) using the Trizol reagent (Ambion, Life Technologies, California, USA), and RNA-seq libraries were prepared according to the manufacturer’s protocol (Illumina Inc. San Diego, CA, USA). The libraries were then sequenced on an Illumina Hiseq 2500 instrument (Illumina, San Diego, CA) to produce 150 bp paired-end reads. qRT-PCR assays were conducted according to our previously described method [22]. Primers were designed using the IDT Primer Quest web tool (http://www.idtdna.com/primerquest/Home/Index). The cDNA was synthesized by Prime Script RT Reagent Kit (Perfect Real Time) (TaKaRa Bio Inc. Dalian, China). The qRT-PCR was performed using GoTaq 2-Step qRT-PCR System (Promega, Madison, WI, USA) and on the ABI Prism1 7500 (Applied Biosystems, Carlsbad, CA, USA). qRT-PCR was performed with the follow PCRcycling condition: 95 °C for 10 s, followed by 40 cycles of 95 °C for 30 s and 60 °C for 1 min. Two housekeeping genes, actin (TRINITY_DN78316_c0_g1) and Alpha tubulin (TRINITY_DN106409_c0_g1), were selected to use as reference genes for normalization of gene expression in the qRT-PCR analysis. The relative expression levels of each gene were evaluated by the comparative2^-ΔΔCt^.

### 4.3. Bioinformatics Analysis

We first used fastp [23] to remove the reads that contained adaptor sequences, low quality bases, and undetermined bases (Ns). The sequencing quality was then assessed using FastQC (http://www.bioinformatics.babraham.ac.uk/projects/fastqc/). All downstream analyses were based on high quality, clean sequencing read data. De novo assembly of the transcriptome was performed with Trinity 2.4.0 with default parameter [24]. All assembled unigenes were used as queries to search the non-redundant (Nr) protein database of all included species (http://www.ncbi.nlm.nih.gov/) by blastx with a threshold of E <0.00001. RSEM was used to determine the expression levels for unigenes by calculating TPM [25]. The differentially expressed unigenes were selected using the criteria of log2 (fold change) >1 or log2 (fold change) <−1, and the statistical significance (p-adjust value <0.05) was determined using the DEGseq package [26]. Venn diagrams were drawn using an online tool (http://bioinformatics.psb.ugent.be/webtools/Venn). GO and KEGG enrichment analyses were then performed on the differentially expressed unigenes using Goatools [27] and KOBAS software [28], respectively. Benjamini-hochberg correction was applied for GO and KEGG enrichment and significance set to adjusted P-value < 0.05. We used the MapMan tool [29] for a graphical overview of the biotic stress response by default parameters. We used the pfam domains PF01397 and PF03936 to assess terpene synthase (TPS) genes in the *P.* *acerifolia* transcriptome assembly using the HMMER package [30] with a threshold of 0.01. Phylogenetic trees were constructed using the neighbor-joining method as implemented in MEGA6.06 [31] with 1000 bootstrap replicates.

### 4.4. Metabolome Analysis

The leaf samples treatment method is the same as for RNA-seq except for six biological replicates; leaf metabolites were extracted using a previously described method [32]. Briefly, leaf samples were ground to a fine powder in liquid nitrogen, after which they were homogenized in methanol/chloroform and cold water. The extracted metabolites were then derivatized with methoxyamine hydrochloride and N-methyl-N-(trimethylsilyl) trifluoroacetamide (Sigma-Aldrich, St. Louis Missouri, USA). The instrument used was a 4D GC×GC-TOF-MS (LECO, San Jose, USA) with DB-5MS capillary column (30 m × 250 μm × 0.25 μm, J&W Scientific, Folsom, CA, USA). One microliter per sample was injected using a splitless model. With helium as the carrier gas, the gas flow rate through the column was 1 mL/min. The following temperature program was used, 2 min at 60 °C, increase to 200 °C with 5 °C /min, then increase to 300 °C with 10 °C min /min, and held 5 min. The temperature of injection, transfer line, and ion source was 250, 280, and 250 °C, respectively. Mass spectrometry used full scan mode with m/z range from 35 to 600, 3-min solvent delay. Data processing and analysis used Chroma TOF 4.3X software (LECO Corporation, St. Joseph, MI, United States). Compounds were identified by searching against the LECO/Fiehn metabolite Mass spectral library. Concentrations of tested metabolite were corrected with an internal standard, arabinose. PCA and PLADA were analyzed using the web tool metaboanalyst (https://www.metaboanalyst.ca) [33]. The metabolite quantities were compared by ANOVA in IBM SPSS Statistics software 20 [34]. The metabolic and transcriptomic data were integrated with the KEGG pathway mapping. Heat maps were drawn with R.

## Figures and Tables

**Table 1:** Output RNA-seq statistics from leaf samples of *P. acerifolia.*

Sample	Raw Bases	Clean Bases	Q20 (%)	Q30 (%)
CK24h_1	8.66 × 10^9^	8.46 × 10^9^	97.91	93.70
CK24h_2	8.24 × 10^9^	8.06 × 10^9^	98.10	94.13
CK24h_3	7.51 × 10^9^	7.35 × 10^9^	97.76	93.31
CK48h_1	8.18 × 10^9^	8.00 × 10^9^	97.68	93.17
CK48h_2	7.08 × 10^9^	6.94 × 10^9^	98.22	94.41
CK48h_3	8.45 × 10^9^	8.25 × 10^9^	97.89	93.64
T24h_1	8.00 × 10^9^	7.81 × 10^9^	97.71	93.22
T24h_2	7.55 × 10^9^	7.40 × 10^9^	98.13	94.20
T24h_3	8.15 × 10^9^	7.97 × 10^9^	97.99	93.87
T48h_1	8.41 × 10^9^	8.23 × 10^9^	97.92	93.70
T48h_2	8.09 × 10^9^	7.90 × 10^9^	97.84	93.52
T48h_3	7.92 × 10^9^	7.75 × 10^9^	98.14	94.21

**Table 2:** Statistics for the transcriptome assemblies derived from *P. acerifolia* leaf RNA.

Type	Transcripts num	Unigenes num	Total Sequence Base	Largest	Smallest	Average Length	N50	Mean Mapped Reads
Resource	199080	121136	2.02 × 10^8^	18931	201	1015.15	1579	1442.764

## Supplementary Materials

The following are available online at https://www.mdpi.com/1422-0067/20/14/3465/s1.
