# Supplementary figures and images for: Changes in Gene Expression and Metabolite Profiles in Platanus acerifolia Leaves in Response to Feeding Damage Caused by Corythucha ciliata
Source: Int J Mol Sci. 2019 Jul 15;20(14):3465. doi: 10.3390/ijms20143465 (PMC6678411; doi:10.3390/ijms20143465)

GO enrichment analysis(CK24h\_vs\_T24h\_G)

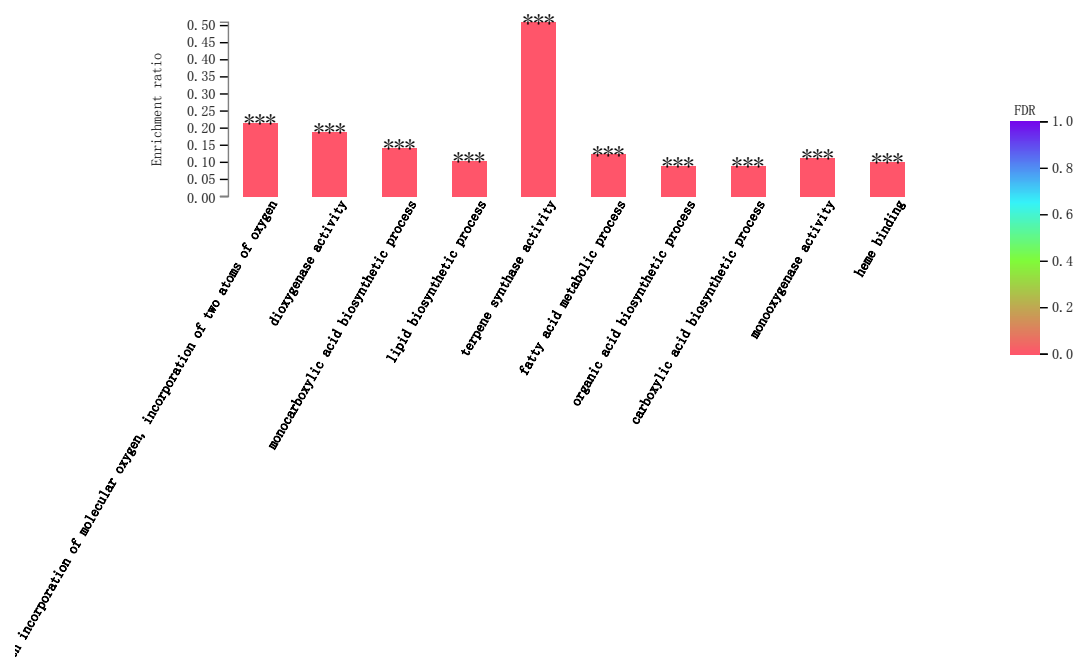

Supplement: Supplementary file 1 [file ijms-20-03465-s001.zip › Supplementary File/FigS1.pdf]

GO enrichment analysis(CK48h\_vs\_T48h\_G)

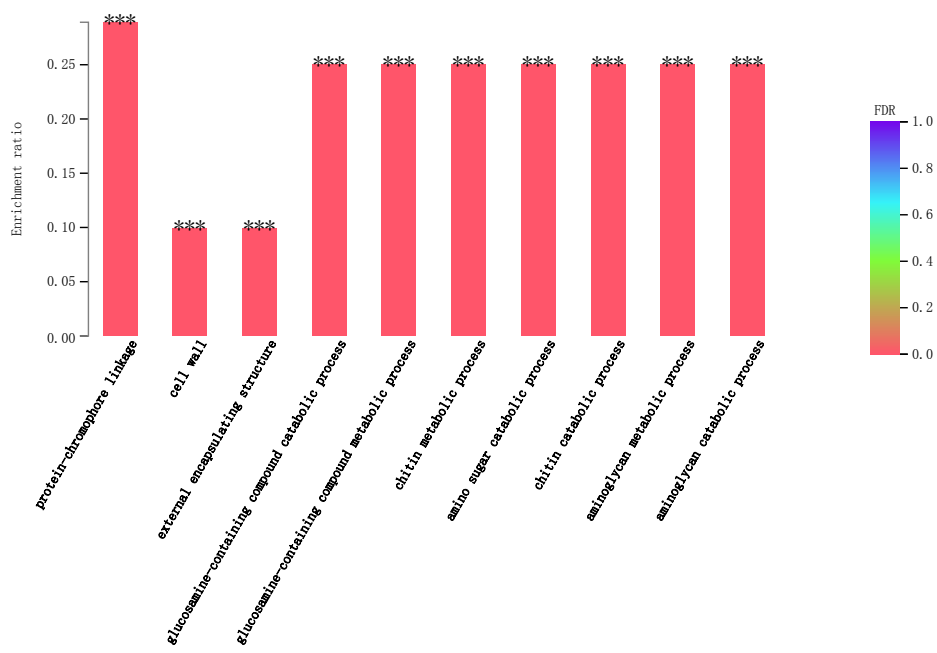

Supplement: Supplementary file 1 [file ijms-20-03465-s001.zip › Supplementary File/FigS2.pdf]

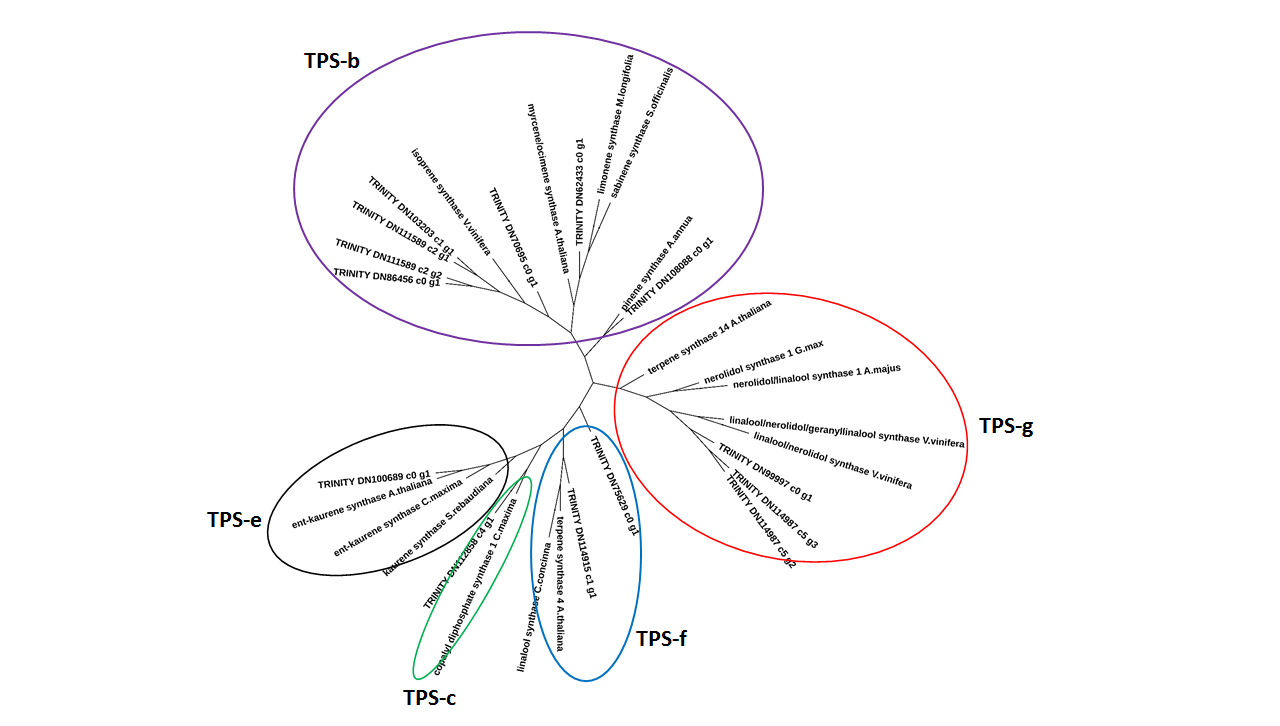

Supplement: Supplementary file 1 [file ijms-20-03465-s001.zip › Supplementary File/FigS3.tif]

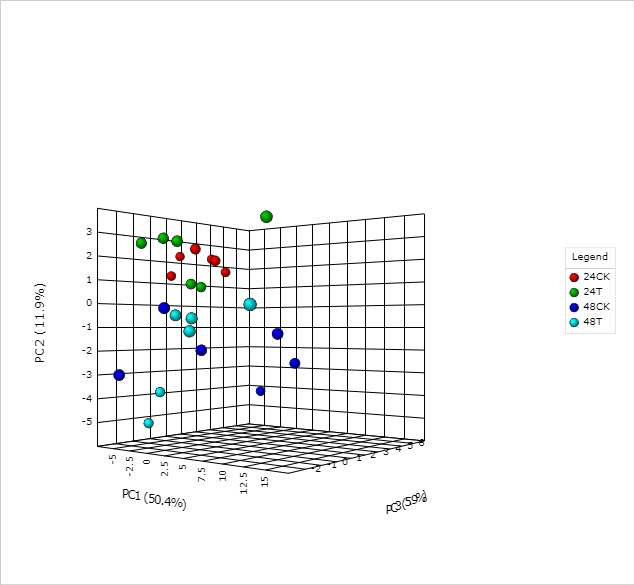

Supplement: Supplementary file 1 [file ijms-20-03465-s001.zip › Supplementary File/FigS4.png]
